# Assessment of aggregate index of systemic inflammation and systemic inflammatory response index in dry age-related macular degeneration: a retrospective study

**DOI:** 10.3389/fmed.2023.1143045

**Published:** 2023-04-25

**Authors:** Naif S. Sannan

**Affiliations:** ^1^Department of Clinical Laboratory Sciences, College of Applied Medical Sciences, King Saud bin Abdulaziz University for Health Sciences, Jeddah, Saudi Arabia; ^2^Biomedical Research Department, King Abdullah International Medical Research Center, Jeddah, Saudi Arabia

**Keywords:** AMD, retina, macula, inflammation, biomarker, AISI, SIRI

## Abstract

**Introduction:**

Inflammation is known to contribute to the development of age-related macular degeneration (AMD). Several inflammatory indices derived from routine complete blood counts have been proposed as biomarkers in multiple disorders.

**Methods:**

In this study, clinical and laboratory data were retrospectively collected from medical records to assess the aggregate index of systemic inflammation (AISI) and the systemic inflammatory response index (SIRI) as potential biomarkers of systemic inflammation in patients with early diagnosis of dry AMD.

**Results:**

The study included 90 patients with dry AMD and 270 age/sex-matched patients with cataracts as a control group. There were no significant differences in the AISI and SIRI results between the cases and controls (*p* = 0.16 and 0.19, respectively).

**Conclusion:**

This suggests that AISI and SIRI may be inadequate metrics for AMD or lack sensitivity in detecting inflammatory changes. Exploring other routine blood markers may help to identify and prevent the early stages of AMD.

## Introduction

Age-related macular degeneration (AMD) is a chronic, progressive degenerative disease of the retina that is characterized by the loss of central vision. It is a leading cause of visual impairment and blindness in older adults (1). AMD is divided into early-stage, characterized by the presence of drusen and changes in the retinal pigment epithelium, and late-stage, which can be either neovascular (wet or exudative) or non-neovascular (atrophic, or non-exudative) (2).

Systemic inflammation may contribute to AMD development and progression, as some inflammatory diseases share risks and an inflammatory profile with AMD (3). The exact mechanisms by which inflammation contributes to the development of AMD are not fully understood, but it is thought to involve the production of reactive oxygen species and other oxidative stress-related factors that can damage the retina and contribute to the development of AMD. In addition, inflammation may stimulate the production of pro-angiogenic factors, which can promote the growth of new blood vessels in the retina and increase the risk of wet AMD (4, 5).

The examination of blood components, including inflammatory indices and genetic variations, has the potential to facilitate the early detection of AMD. For instance, elevated levels of inflammation-related biomarkers, such as C-reactive protein, lipids, and interleukin-6, have been found in patients with AMD compared to those without the disease (6–9). Certain genetic variations have also been linked to increasing the risk of developing AMD (10, 11). An elevated white blood cell count was linked to an increased risk of developing AMD (12).

Systemic inflammatory indices derived from complete blood count (CBC) tests, including the neutrophil/lymphocyte ratio (NLR), derived-NLR, platelet/lymphocyte ratio (PLR), monocyte/lymphocyte ratio (MLR), have received attention in recent years due to their low cost, accessibility, and predictive power for outcomes in several disorders including AMD (13–17).

The systemic inflammatory response index (SIRI) is calculated by multiplying the neutrophil and monocyte counts and then dividing the product by the lymphocyte count. Variations in SIRI values have been shown to be associated with clinical outcomes in a variety of cancers, including pancreatic (18), gallbladder (19), gastric (20), breast (21), and cervical cancers (22), as well as in COVID-19 patients (23). Similarly, the aggregate index of systemic inflammation (AISI) is calculated by multiplying the counts of neutrophils, monocytes, and platelets and then dividing the product by the lymphocyte count. While AISI has been studied relatively sparingly, recent research has investigated its relationship to neoplastic conditions such as non-small-cell lung cancer (24) and COVID-19 (23). The cells involved in both AISI and SIRI calculations are critical in maintaining a well-balanced immune system, which helps protect the body from harmful pathogens and diseases. However, it’s important to note that these cells can also produce pro-inflammatory substances that have been associated with various inflammatory diseases (25, 26).

This study aimed to assess the value of two CBC-derived indices, AISI and SIRI, in dry AMD since their diagnostic role remains to be elucidated. A literature search has revealed one study that assessed the levels of AISI and SIRI in individuals with AMD and was conducted on a cohort of men with Sardinian ancestry (27).

## Methods

This study represents a secondary analysis of previously reported patients’ data (28). Briefly, Electronic health records at a tertiary care hospital (King Abdulaziz Medical City, Jeddah, Saudi Arabia) were enquired for new AMD patients (aged ≥50 years) with macular drusen in at least one eye, with or without signs of geographic atrophy, and other fundus characteristics. Exclusion criteria included wet AMD in the fellow eye, inflammatory ocular disorders, other ophthalmic conditions, malignancies, hematological and autoimmune disorders, chronic inflammatory disorders, leukocytosis (>11 × 10^3^ cells/mm^3^), leukopenia (<4 × 10^3^ cells/mm^3^), thrombocytosis (>450 × 10^3^ cells/mm^3^), and thrombocytopenia (<150 × 10^3^ cells/mm^3^). CBC results [Cell-Dyn Sapphire (Abbott Diagnostics Division, Santa Clara, CA)] were collected for cases and controls, and the AISI ((neutrophils × monocytes × platelets)/lymphocytes) and SIRI ((neutrophil × monocytes)/lymphocytes) ratios were calculated. Informed consent was obtained from all participants, and the study was conducted following the Declaration of Helsinki. Institutional review board approval was granted by the King Abdullah International Medical Research Center (#RJ20/106/J-SP21J/083/03). The control group for the study was composed of cataract individuals without a previous diagnosis of AMD. The sample size of the control group was determined to be three times the size of the AMD group, to increase the accuracy and statistical power of the study and reduce the potential for selection bias. Control subjects were randomly selected from the electronic health records, and their ages were closely matched to the mean age of the AMD group (within a range of 5 years). The chi-square test and *t*-test were used to analyze differences in demographics and blood result characteristics between the case and control groups. Multivariate analysis was conducted, taking into account age and sex, to further examine differences between the groups. The assumptions of the linear relationship and homogeneity of the regression slopes were checked and all models met these assumptions. All statistical analyses were performed using SAS software (version 9.4, SAS Institute Inc.), with *p*-values and confidence intervals at 95%.

## Results

The study included 90 participants in the case group (22.55% of the total sample) and 270 participants in the control group (77.44%). The demographics and hematological parameters of the case and control groups are presented in Table 1. In the univariate analysis, the mean AISI for the case group was 471.18 ± 163.8 and 477.32 ± 239.5 for the control group. The mean SIRI for the case group was 1.99 ± 0.94 and 1.84 ± 0.93 for the control group. There were no statistically significant differences between the two groups (Table 1).

**Table 1:** Demographic and hematological parameters of dry age-related macular degeneration (AMD) cases and controls.

	Dry AMD *n* (%)^1^	Controls *n* (%)	*p*-value cases vs. controls^2^
Total^3^	90 (22.55)	270 (77.44)	–
Age (yr), mean ± SD^3^	71 ± 9	70 ± 7	0.27
Sex^3^			0.63
Male^3^	41 (45.56)	132 (42.72)	
Female^3^	49 (54.44)	177 (57.28)	
Hematological parameters	Mean ± SD		
Neutrophils (10^9^/L)^3^	4.55 ± 3.73	4.2 ± 3.84	0.79
Monocytes (10^9^/L)^3^	0.63 ± 0.26	0.602 ± 0.21	0.63
Platelets (10^9^/L)^3^	268.61 ± 83.98	268.73 ± 100.65	<0.0001^*^
Lymphocytes (10^9^/L)^3^	2.29 ± 1.12	2.39 ± 0.98	0.44
AISI^4^	471.18 ± 163.8	477.32 ± 239.5	0.74
SIRI^5^	1.99 ± 0.94	1.84 ± 0.93	0.75

SD, standard deviation. ^*^Calculated from log transformation of variables.

1 “*n*” sample size, % percentage.

2 Chi-square test for categorical variables and *t*-test for numeric variables.

3 Reference (28).

4 Aggregate index of systemic inflammation = (neutrophils × monocytes × platelets)/lymphocytes.

5 Systemic inflammatory response index = (neutrophil × monocytes)/lymphocytes.

In the regression analysis (adjusted for age and sex, Table 2), the AISI was not significantly associated with the development of AMD compared to controls, with a beta coefficient of 0.16 (standard error 0.11, *p* = 0.16). Similarly, the SIRI was not significantly associated with the development of AMD compared to controls, with a beta coefficient of 0.14 (standard error 0.11, *p* = 0.19). These results suggest that AISI and SIRI are not significant predictors of AMD. In addition, these models showed that age and sex had no significant effects on the AISI and SIRI values in the case group compared to the control group.

**Table 2:** Regression analysis to predict factors associated with age-related macular degeneration (AMD) compared to controls.

Hematological parameters	Dry AMD	Controls	*p*-value cases vs. controls^1^
	Beta coefficient	Standard error	
Neutrophils^2^	−0.02	0.05	0.61
Monocytes^2^	0.001	0.02	0.96
Platelets^2^	−0.03	0.04	0.96
Lymphocytes^2^	0.05	0.04	0.21
AISI^3^	0.16	0.11	0.16
SIRI^4^	0.14	0.11	0.19

The analysis was adjusted for age and sex.

1 Analysis of covariance used to estimate differences in covariances across patients with dry AMD and controls, adjusted for age and sex, and the Kruskal Wallis test for non-normal numeric variables.

2 Reference (28).

3 Aggregate index of systemic inflammation = (neutrophils × monocytes × platelets)/lymphocytes.

4 Systemic inflammatory response index = (neutrophil × monocytes)/lymphocytes.

## Discussion

Although AISI is thought to be a more precise indicator of inflammation than other indices that only consider fewer cell types, it has not been widely used or studied in the literature. The AISI index was evaluated in several conditions such as a prognostic marker for small cell lung carcinoma (29), identification of patients at risk of prolonged hospital stay in open elective thoracic surgery (30), and as a predictor for severity and intensive care unit admission in COVID-19 patients (31). Another study has highlighted the prognostic significance of AISI in predicting poor outcomes in patients with idiopathic pulmonary fibrosis (32). AISI is a measure of inflammation that takes into account several different types of cells involved in the immune response, including neutrophils, lymphocytes, and platelets, as well as the monocyte count. These cells play a role in producing proinflammatory substances such as cytokines, chemokines, enzymes, and reactive oxidative species, which can contribute to inflammation and the development of certain diseases (25). SIRI is another immune system biomarker that has been studied in various disorders. The SIRI index was first introduced by Qi et al. in a study of pancreatic cancer patients, where it was shown to be a useful predictor of prognosis (33). The SIRI is a measure that considers three types of white blood cells, neutrophils × monocytes/lymphocytes, to provide information about the overall balance of immune and inflammation activity in the body (33). Additional studies have shown that the SIRI may be able to predict survival in several types of cancer, including pancreatic cancer (18), gallbladder cancer (19), oral squamous cell carcinoma (34), and cervical cancer (22).

The purpose of this study was to assess the ability of the AISI and SIRI to detect AMD in the early stage of disease development. However, there were no significant differences in AISI and SIRI results between the case and control groups. This suggests that the metrics of AISI and SIRI may be inadequate in accurately assessing the inflammatory changes that are associated with AMD, or they may exhibit insufficient sensitivity in detecting such changes. The findings of this study are in line with those of a recent publication on AMD patients of Sardinian ancestry (27), which appears to be the only study on AISI and SIRI in AMD.

Previous studies have yielded conflicting results on the association between white blood cells and AMD, with some studies finding correlations between higher counts and increased risk of AMD (12, 35) while others did not (36, 37). More recent studies have looked at various blood components derived from routine CBC measurements to calculate inflammatory relevant indices in AMD patients. For instance, studies have reported higher levels of neutrophils and lower levels of lymphocytes in AMD patients compared to controls (27, 28, 38). Furthermore, it has been demonstrated that the NLR and MLR are significantly elevated in patients with wet AMD compared to individuals with dry AMD and healthy controls (39), with ratios indicating low-grade inflammation. These observations strongly suggest that alterations in CBC parameters could serve as potential markers of AMD-related inflammation. Neutrophils are the most abundant type of white blood cell in the body and play a role in acute and chronic inflammation, phagocytosis, and the release of anti-inflammatory mediators (40, 41). Lymphocytes are involved in both the initiation and resolution of inflammation and may be activated or suppressed in response to various signals. Lymphocyte infiltration plays a role in the initiation and progression of inflammatory responses, a key contributor to the tissue damage and functional impairment that occur in inflammatory disorders (41, 42). Monocytes are a type of white blood cell that is also involved in the immune response and inflammation (41, 43). A high PLR is considered a negative prognostic factor for inflammatory diseases, as an elevated platelet count can result in lymphopenia (44, 45). It is possible that neutrophils and lymphocytes play a more crucial role in the development of AMD than other cell types, and their influence could be “diluted” by taking into account multiple cell types in AISI and SIRI calculations. Additionally, it is also plausible that local inflammation in the retina may not be accurately reflected by systemic inflammatory indices, leading to an apparent disconnect between the presence of certain cell types and systemic inflammatory markers.

This study has some limitations that should be taken into account when interpreting the results. One of these limitations is the relatively small sample size, which may not be representative of the entire population and could lead to insufficient statistical power to detect certain trends or associations. Additionally, the study is retrospective in design, meaning that it looks back at past data and events, which may be subject to bias or error. Finally, the study was conducted at a single center, so the results may not be generalizable to other settings or populations. In addition, the control group in this study consists of cataract patients, which may have biased the results of the CBC analysis. A literature search revealed no prior studies regarding the association of cataractogenesis with AISI and SIRI. Also, it should be noted that inflammatory pathologies may play a role in the onset of cataracts (46, 47) and could also be associated with post-cataract surgery complications (48). This could have affected the accuracy of the AISI and the SIRI as potential biomarkers for the early detection of AMD. Therefore, it is important to consider these limitations when evaluating the findings of this study, and it would be beneficial for future research to confirm and build upon these results. Further research is needed to elucidate the underlying mechanisms that link immune-related cell types with AMD and to explore the potential limitations of using systemic inflammatory response indices to assess ocular inflammatory conditions.

## Conclusion

This study found that the AISI and the SIRI are not effective biomarkers for the early detection of AMD. These results suggest that further research is needed to identify other potential biomarkers that can be used to identify and prevent the early stages of AMD.

## Institutional review board statement

The study was conducted according to the guidelines of the Declaration of Helsinki, and approved by the Institutional Review Board of King Abdullah International Medical Research Center (protocol code# RJ20/106/J-SP21J/083/03).

## Data availability statement

The raw data supporting the conclusions of this article will be made available by the authors, without undue reservation.

## Ethics statement

The studies involving human participants were reviewed and approved by King Abdullah International Medical Research Center Ethics Board. The patients/participants provided their written informed consent to participate in this study.

## Author contributions

The author confirms being the sole contributor of this work and has approved it for publication.

## Conflict of interest

The author declares that the research was conducted in the absence of any commercial or financial relationships that could be construed as a potential conflict of interest.

## Publisher’s note

All claims expressed in this article are solely those of the authors and do not necessarily represent those of their affiliated organizations, or those of the publisher, the editors and the reviewers. Any product that may be evaluated in this article, or claim that may be made by its manufacturer, is not guaranteed or endorsed by the publisher.
